# Electrochemical biostimulation of aerobic metabolic TCE degradation in a bioaugmentation approach

**DOI:** 10.1007/s11356-023-29839-1

**Published:** 2023-09-22

**Authors:** Steffen Hertle, Nick de Boni, Heico Schell, Andreas Tiehm

**Affiliations:** TZW:DVGW Technologiezentrum Wasser, Department Water Microbiology, Karlsruher Straße 84, 76139 Karlsruhe, Germany

**Keywords:** Bioaugmentation, Bioremediation, Aerobic metabolic biodegradation, Trichloroethene, Electrochemical biostimulation

## Abstract

Chloroethenes are globally prevalent groundwater contaminants. Since 2014, TCE has been shown to be degradable in an aerobic metabolic process where it is used as sole energy source and growth substrate by a mixed bacteria culture (SF culture). In 2019, the SF culture was shown to be successfully used in bioaugmentation approaches under field-relevant conditions. In this study, a combined bio-/electro-approach to stimulate the TCE degradation by the SF culture was investigated in laboratory experiments. Column experiments were set up to compare a bioaugmentation approach with an electrochemical biostimulated bioaugmentation approach. Low strength direct current increased the amount of degraded TCE to about 150 % of the control. Through lowering the inflow concentration of oxygen, the effect of the electro-biostimulation in a low oxygen setting confirmed the potential of the bio-electro process for treatment of oxygen-deprived, TCE-contaminated sites.

## Introduction

Chlorinated ethenes (CEs) have been used as degreaser in the metal industry as well as dry cleaning solvents. Through accidental spills, as well as improper handling, the solvents find their way into the environment. CEs are some of the most common groundwater contaminants worldwide (Conrad et al. 2010). The CE tetrachloroethene (perchloroethene (PCE)), trichloroethene (TCE), cis-1,2-dichloroethene (cDCE) and vinylchloride (VC) are part of the US priority list of hazardous substances (ATSDR 2019) due to their toxic and carcinogenic properties. In comparison with their unsubstituted hydrocarbons, CEs are more recalcitrant to biodegradation (Lyon and Vogel 2013).

Biological degradation of the higher chlorinated ethenes PCE and TCE is primarily achieved by anaerobic, reductive dechlorination, while aerobic processes become feasible for the less chlorinated ethenes cDCE and VC. Both anaerobic and aerobic processes take part in bioremediation of chloroethenes, and have proven to be a viable method for the remediation of CE-contaminated sites (Tiehm and Schmidt 2011).

During complete reductive dechlorination, PCE and TCE are transformed to ethene, with cDCE and VC as stable intermediate products. While various bacteria are able to transform PCE and TCE to cDCE and VC, only bacteria of the species *Dehalococcoides* spp. are known to be capable of performing complete dechlorination of the contaminants to ethene under anaerobic conditions. Therefore, without the presence of *Dehalococcoides* and suitable hydrochemical conditions, the reductive dechlorination can stall at cDCE and VC, resulting in accumulation of the hazardous intermediate products.

While the higher chlorinated CE are prone to undergo reductive dechlorination, in heavily reduced aquifers without other terminal electron acceptors like oxygen, nitrate and sulfate (Illman and Alvarez 2009), the lower CEs are degraded faster under aerobic conditions (Bradley 2003; Lohner et al. 2011). While there is, to our knowledge, no known aerobic metabolic degradation process for PCE, cases of co-metabolic aerobic degradation of PCE (Ryoo et al. 2000) and TCE (Dey and Roy 2009; Mattes et al. 2010) as well as aerobic metabolic degradation of TCE have been shown (Schmidt et al. 2014; Gaza et al. 2019; Willmann et al. 2023).

Co-metabolic degradation of CE happens fortuitously by enzymes (oxygenase) induced by the bacterial growth substrate, which is used as the auxiliary substrate. Various molecules like ammonium (Sayavedra-Soto et al. 2010), methane (Hazen et al. 2009), toluene and phenol (Shingleton et al. 2001) and VC (Tiehm et al. 2008) can be used as auxiliary substrate. The competition of the CE and the auxiliary substrate for the active site of the oxygenase limits the efficiency of the remediation process. Furthermore, reactive epoxides released during co-metabolic degradation are not further metabolized, causing toxic effects, which can inhibit the CE degradation (Mattes et al. 2010).

Aerobic metabolic degradation of TCE offers various advantages over reductive dechlorination as well as aerobic co-metabolic processes. Due to its metabolic nature, the addition of auxiliary substrates is not necessary, therefore enabling the available oxygen to be utilized exclusively for the biodegradation purpose, increasing the overall efficiency of the remediation approach. Schmidt et al. (2014) demonstrated the aerobic metabolic TCE degradation with a mixed bacterial culture (SF culture). Bacteria growth, stoichiometric chloride formation and stable carbon isotope fractionation with TCE as single carbon source in the absence of any known auxiliary substrate were shown.

Since, to our knowledge, aerobic metabolic TCE degradation has only been shown on a few other sites contaminated with CEs (Willmann et al. 2023), using the SF culture in a bioaugmentation approach is of special interest. Gaza et al. (2019) demonstrated the viability of a bioaugmentation approach using the SF culture under field relevant conditions, enabling TCE degradation in lab experiments with different, CE-contaminated groundwater, where TCE degradation was not seen naturally.

Most contaminated sites turn anoxic over time, due to aerobic microbial processes and the presence of reduced organic carbons. Due to a surplus of contaminants, the oxygen replenished by the groundwater flow is immediately consumed in the degradation of contaminants, leaving the downstream area of the site in anoxic conditions. In order to enhance the aerobic degradation of contaminants, oxygen needs to be delivered into the bio-reactive zone of the site.

This study focuses on a combined electrochemical/microbiological approach for TCE degradation. While TCE degradation is achieved through microbiota, required oxygen is replenished through hydrolysis.

## Material and methods

### Bacteria culture

A mixed bacteria culture (SF culture) capable of aerobic metabolic TCE degradation was used for the experiments. The culture was maintained in mineral salt medium (MSM) with TCE as sole carbon source without any known auxiliary substrates (Schmidt et al. 2014; Gaza et al. 2019).

The SF culture contains bacteria of the family of the *Burkholderiaceae* (*Ralstonia* spp*.*), *Comamonadaceae* (*Polaromonas* spp.) and *Rhodocyclaceae*. Further characterization of the mixed bacteria culture on a species level as well as the contribution of the different species to the TCE degradation is still under investigation.

### Chemicals

The experiments were conducted with TCE as sole contaminant. TCE used was of 99.9% purity (Fluka).

TCE has a low solubility in water (1 g/L at 20 °C; log*K*_OW_=2.42) and is highly volatile (vapor pressure = 77.6 hPa, evaporation number = 3.8) (IFA 2023).

### Analytical methods

Chloroethenes were measured with an Agilent Technologies (Waldbronn, Germany) 7890A GC system equipped with electron capture detector (ECD) and flame ionization detector (FID) and 7697A headspace sampler. Chloroethenes were measured in the aqueous phase as described (Tiehm et al. 2008). The limit of quantification for TCE is 0.7 μg/L; the limit of detection is 0.2 μg/L. Values represent the average of duplicate measurements, and standard error of the analysis is 10%. For quality control, external standards have been analysed with each run. Recovery rate of the standards was between 95 and 105%.

The chloride concentration was measured using a Metrohm 761 compact ion chromatograph (Filderstadt, Germany) equipped with a conductivity detector and a MetrosepA-Supp-5 column (Zhao et al. 2010; Gaza et al. 2019) with a limit of quantification of 1 mg/L and a limit of detection of 0.3 mg/L. Standard error of the analysis is 5%. Sample analysis was accompanied by the analysis of external standards for quality control. Recovery rate of the standards was between 95 and 105 %.

Free chlorine and total chlorine were measured using the photometric test kit NANOCOLOR Chlorine/Ozone 2 (Macherey & Nagel, Germany).

Dissolved oxygen (DO) and pH were measured with WTW Multi 3620 IDS with FDO 925 and Sentix-940-3-probes.

The amount of oxygen produced by electrolysis of water was calculated according to Faraday’s law of electrolysis.

### Column setup

During the experiment, three columns were used to investigate the electrochemical support of microbial aerobic metabolic TCE degradation. The properties of the different columns are summarized in Table 1. Column “Bio 1” is a bioaugmentation setup without further stimulation. Column “Bio 2” follows a bioaugmentation approach with electrochemical biostimulation through oxygen production by electrolysis of water. The column “Abiotic” is used as control experiment for possible electrochemical degradation or volatilization of TCE. Therefore, the column is equipped with electrodes, similar to Bio 2, but the silica sand has not been inoculated with the SF culture after sterilization. Columns Bio 1 and Bio 2 are fed by the same reservoir, while column Abiotic is fed through a separate reservoir to avoid bacteria growing back through the tubing resulting in the contamination of the abiotic column.

**Table 1:** Properties of the columns operated; Bio 1 and Bio 2 are fed through the same reservoir

Column	Bioactive	DC application	Column length (cm)	Column diameter (mm)	Electrode distance (cm)
Bio 1	X	-	28	35	-
Bio 2	X	X	28	35	10
Abiotic	-	X	28	35	10

A constant volume flow of 0.5 L/day was maintained by a peristaltic pump (Ismatec Masterflex). All connections were made of glass and Viton tubes.

The columns were kept in the dark for the whole experiment duration.

### Immobilization of bacteria on silica sand

In order to immobilize the bacteria culture on silica sand, it was recirculated through two borosilicate glass columns filled with sterile silica sand for 10 weeks. The columns were set up in parallel and fed through the same reservoir. During recirculation, oxygen was provided by ambient air in the headspace of the reservoir containing MSM, the bacteria culture and TCE. TCE was re-dosed when completely degraded to avoid starvation periods during the immobilization phase. Oxygen was replenished by exchanging the ambient air in the reservoir after sampling.

### Flow-through phase

**Fig. 1:**
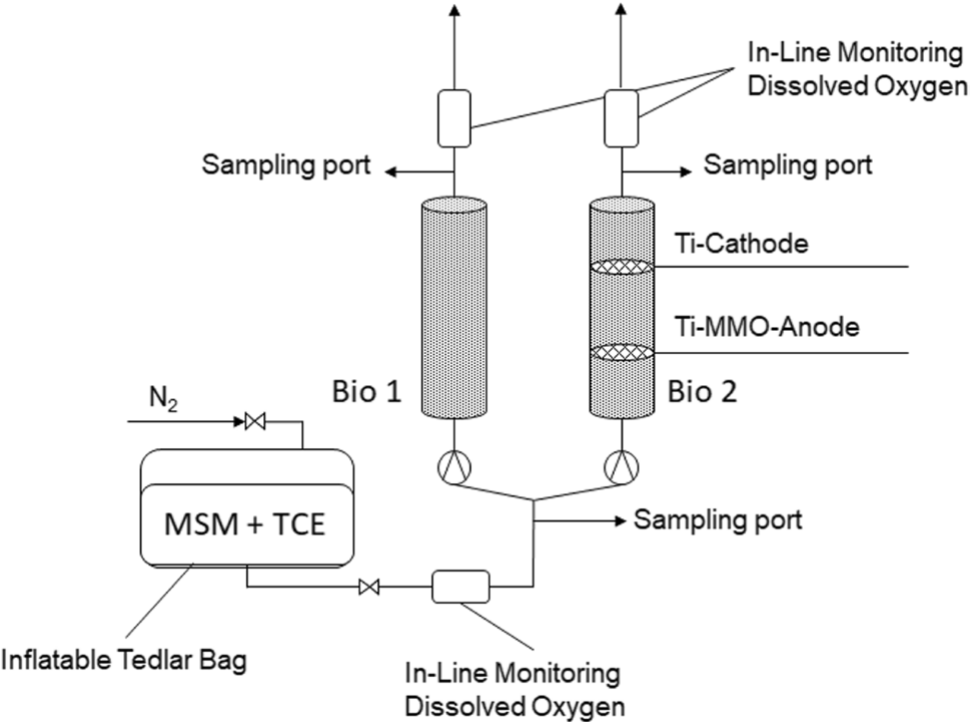
Scheme of the flow-through setup of the columns

After the immobilization period, the columns were adjusted to allow a flow-through scheme (Fig. 1) with a single passage of TCE-spiked, oxygen-saturated, MSM. TCE concentration was periodically increased until the adjusted TCE concentration was sufficient to

- Cause complete consumption of the oxygen available at the inflow (~ 8 mg/L)
- Achieve outflow concentrations of ~ 1/3 of the inflow concentration at a volume flow of ~ 0.5 L/day.

After meeting the criteria given, direct current (DC) was applied to column Bio 2.

### Application of direct current

For the application of DC to support the microbial degradation through electrochemical processes in Bio 2 as well as the abiotic control, a DC power source (HEIDEN Electronics, Germany) was attached to titanium cathodes and mixed metal oxide (MMO)-coated titanium anodes (Metakem, Germany) that are in direct contact with the silica sand bed. Both electrodes were in mesh type B and circular shape with a diameter of 35 mm. Platinum wires were used to connect the electrodes to fittings for banana plugs further connecting to the power supply. The platinum wires were pierced through PTFE septa, sealing the columns. Electro-support started with 0.25 mA DC (~ 2.28 mA/dm^2^) and was adjusted to 0.5 mA (~ 4.56 mA/dm^2^) in the course of the experiment to increase the amount of produced oxygen. DC was monitored with Agilent KeySight Digital Multimeters.

### DC supported aerobic metabolic TCE degradation in oxygen-depleted MSM

To evaluate the viability of the biostimulation by DC in an oxygen-depleted environment, the MSM was fed through a membrane reactor, described by Arcangeli and Arvin (1995), to lower the concentration of the DO. DO concentration of the inflow was kept at or below ~ 3 mg/L in all three columns. This enabled column Bio 1 to continue with TCE degradation, but also increased the possible effect of the electrochemical stimulation in column Bio 2.

In order to evaluate the process under complete anoxic conditions, inflow oxygen was kept at < 0.5 mg/L for the final experiment phase.

In order to evaluate the effect of the DC strength used on TCE in MSM, the abiotic column had 0.5 mA and 0.75 mA (~ 6.84 mA/dm^2^) DC applied.

## Results and discussion

### Application of direct current to oxygen-saturated medium

Once inflow TCE concentration was adjusted to ~ 30–33 mg/L (~ 228–251 μmol/L) over a period of 70 days resulting in outflow concentrations of ~ 10 mg/L and complete oxygen reduction, 0.25 mA DC current was applied to column Bio 2 in order to increase the amount of available oxygen. By applying 0.25 mA DC, 3.6 mg/L additional oxygen is available in the supported column leading to a total amount of ~ 11.6 mg/L DO available in Bio 2. With a stoichiometric degradation of TCE (utilization factor equals 2.7 mg (TCE)/mg (O_2_)), ~ 31 mg/L of the contaminant can be degraded, resulting in the TCE-outflow concentration of up to 2 mg/L. Increasing the current to 0.5 mA DC after 96 days, leading to an additional DO amount of 7.2 mg/L, resulted in TCE outflow concentrations of ~ 0.01 mg/L and a surplus of oxygen in the outflow of around 1 mg/L. For the given TCE-inflow concentration of 30–33 mg/L, stoichiometric required DO concentration is at 11.1–12.2 mg/L leaving ~ 3 mg/L of surplus oxygen calculated. Measured DO at the outflow of Bio 2 was at ~ 1.5–2 mg/L during the DC application of 0.5 mA. The production of 7.2 mg/L DO at 0.5 mA and the 3.6 mg/L DO at 0.25 mA is assuming 100 % energetic efficiency of the electrolysis process with no side reactions.

**Fig. 2:**
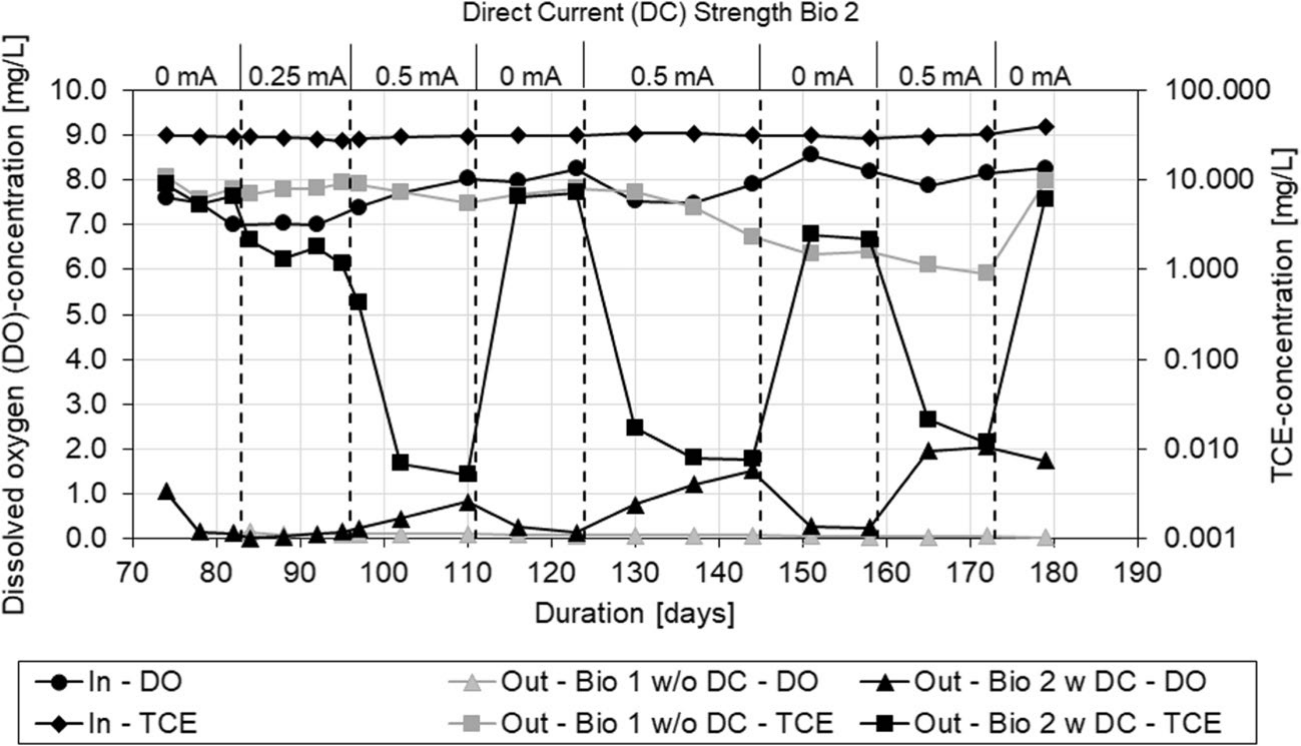
Inflow and outflow concentration of DO and TCE of the bioaugmented columns after the initial startup phase, during the electrochemical biostimulation in the timespan of 70–180 days of experiment duration. The electric current was varied to demonstrate biostimulation by water electrolysis. Standard error (σ_n_) of the TCE analysis is 10% and 1.5% for the DO measurement. The error bars are completely covered by the corresponding markers

By switching off the DC for 2 weeks after 110 days and 144 days, the degradation in column Bio 2 reverted back to ~ 20 mg/L indicating that the electrochemical support did not harm the microbiota established in the column. Reapplying the 0.5 mA resulted in enhanced degradation of the inflow TCE, clearly linking the increased performance of TCE degradation to the applied current (Fig. 2).

### Application of direct current to oxygen-deprived medium

During transition from oxygen-saturated MSM to oxygen-deprived MSM, the biotic columns went into a starvation period of 5 weeks with no MSM feeding resulting in anaerobic conditions inside the columns. The DC was detached from Bio 2 during the starvation period to avoid a shift of the pH in the anode/cathode area as well as gas buildup.

**Fig. 3:**
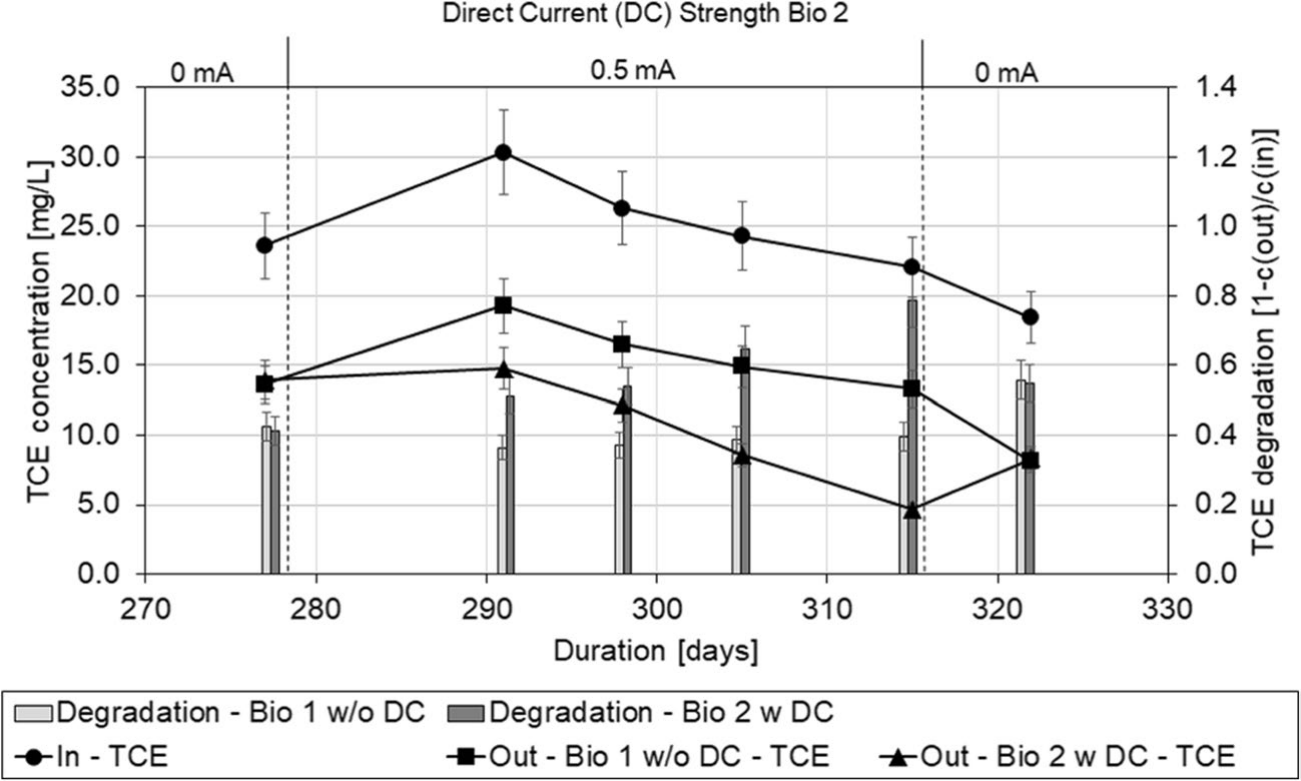
Inflow and outflow concentration of TCE of the bioaugmented columns using oxygen-deprived MSM, during the time span of 270–330 days of experiment duration. Inflow DO concentration was < 1.0 mg/L during the duration shown. Standard error (σ_n_) of the TCE analysis is 10%

During the course of this experiment phase, shown in Fig. 3, TCE degradation became quickly limited by the available DO in the inflow. But even with low amounts of oxygen, a difference of ~ 10 mg/L TCE by column passage was achieved in both columns, which aligns with the chloride produced. With the support of 0.5 mA DC, starting after day 278, degradation of TCE in Bio 2 slowly increased to ~ 15 mg/L, while the outflow concentration of DO increased to ~ 1 mg/L immediately and decreased again with the increasing TCE degradation. While there is no limitation through oxygen or TCE, the degradation process becomes limited by the degradation kinetics or size of the reactive zone, respectively. Detaching the current, at the end of the experiment phase, led the degradation efficiency of Bio 2 to fall and align with Bio 1 again.

In order to rule out electrochemical TCE degradation during the DC-supported phase, inflow and outflow of the abiotic, but electrochemically treated, column was monitored in parallel. While an increase in DO by column passage was measurable using oxygen-deprived MSM, the inflow and outflow concentration of TCE and chloride was stable (data not shown), indicating that, at the electric currents tested, no electrochemical TCE degradation took place. However, starting at 0.75 mA DC, extensive gas production was noticeable.

Therefore, in the biotic columns, TCE decrease that is linked with an increase in chloride can be attributed to aerobic metabolic TCE degradation, and the further increase in Bio 2 is caused by the electrochemical oxygen supply rather than a direct electrochemical degradation of the TCE.

During the whole experiment, the recovery rate of released chloride from the degradation of TCE was at ~ 80 % in both biotic columns. While no stable metabolites were detected with the GC systems, volatilization losses are possible due to the setup of the experiment columns and the high volatility of TCE (vapor pressure = 77.6 hPa, evaporation number = 3.8 (IFA 2023)). The wire connections of the electrodes were pierced through PTFE septa. While no leakage of MSM was detected, abiotic TCE losses by volatilization could still happen through the pierced septa and the various connections of glassware and tubing.

Measurements of free chlorine and total chlorine at inflow and outflow of the columns were below the detection limit of 0.05 mg/L in all columns over the complete duration of the experiment. Measurements of the porewater between the electrodes were not possible; therefore, creation of chlorine with subsequent reduction to chloride at the cathode cannot be ruled out.

The pH of the MSM slightly decreased through column passage in Bio 1 and Bio 2, while it was stable in abiotic. DC application did not affect pH values, hinting that the pH decrease in the bioactive columns was caused by the release of HCl and CO_2_ subsequent to the TCE degradation. For the duration of the experiments, inflow and outflow pH values of all columns were between 6.8 and 7.8 (data not shown) and therefore in a feasible range for aerobic metabolic TCE degradation.

**Fig. 4:**
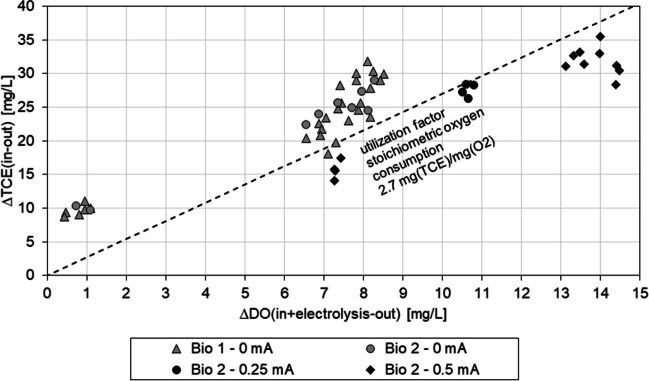
Relation of changes in oxygen and TCE of the bio-active columns. Stoichiometric utilization factor for aerobic metabolic TCE degradation is 2.7 mg (TCE)/mg (O_2_)

When comparing the change in DO with the amount of TCE decreased by column passage, the relation is scattered around the stoichiometric degradation, in which 2.7 mg (TCE)/mg (O_2_) is degraded (Fig. 4). Values for degradation in oxygen-saturated MSM are above the stoichiometric value due to biomass growth. During the EC-supported phases in Bio 2, the values of the degraded TCE are beneath the stoichiometric possible amount. This can be due to the formation of small gas bubbles, resulting in underrepresentation in the amount of DO measured in the outflow. In addition, DO(electrolysis) is calculated based on Faraday’s law of electrolysis and assumes 100 % efficiency of the electrolysis and therefore holds potential for overestimation of the oxygen available.

Due to the possibility of volatilization losses, oxygen diffusion into the columns is possible as well and can contribute to the TCE degradation. Oxygen diffusion into the column system has a bigger impact on TCE degradation under anoxic conditions and can explain the over stoichiometric degradation of TCE during the anoxic phase.

Aulenta et al. (2013) demonstrated the feasibility of an electrolysis-driven process to enhance removal kinetics of a co-metabolic cDCE degradation in batch experiments. Recent developments in bio-electro flow-through systems to treat CEs target TCE by reductive dechlorination while the successor substances cDCE, VC and ethene are oxidized (Lohner et al. 2011; Verdini et al. 2015; Lai et al. 2017; Zeppilli et al. 2019). This study focused on oxidative processes for TCE degradation; therefore, compared to the studies mentioned, the electrode order is reversed, with the anode at the inflow, and the cathode at the outflow of the column system.

A groundwater recirculation setup with periodic current application enabled the aerobic degradation of TCE (Yuan et al. 2021). Since TCE degradation increased by the addition of acetate, the degradation pathway is most likely of co-metabolic nature and therefore reliant on an auxiliary substrate in the form of intrinsic carbons or exogenous growth substrates. Also, ^13^C enrichment factors in the experiment differ from those shown in aerobic metabolic TCE degradation (Schmidt et al. 2014; Gaza et al. 2019) and support the theory that co-metabolic processes are prevalent in the TCE degradation. The same system showed the degradation of TCE in a mixture of chlorinated hydrocarbons in an integrated anaerobic/aerobic approach (Cai et al. 2022).

By utilizing aerobic metabolic TCE degradation, as compared to anaerobic reductive dechlorination, the potential accumulation of toxic metabolites is avoided. Furthermore, as compared to co-metabolic processes, the addition of auxiliary substrates is not necessary, which simplifies the remediation approach since TCE is used as energy source as well as growth substrate. Aside from microbiological degradation, the reduction of TCE can also be accomplished by hydrodechlorination in an electrochemical reaction (Rajic et al. 2015). However, current strength used for electrochemical degradation of chloroethenes (60–180 mA (Rajic et al. 2016; Hyldegaard et al. 2020)) exceeds the current strength used to enhance bioremediation (0.5 mA (this study), 4.3–4.5 mA (Aulenta et al. 2013)). While the abiotic treatment of sinking TCE concentrations increases the current intensity needed in the electrochemical degradation (Hyldegaard et al. 2020), the presented combined bio-electro approach has a stable low energy demand. Nonetheless, abiotic degradation processes can be a feasible option for sites unsuitable for biodegradation.

## Conclusion

A highly active continuous flow column system was set up successfully. Without further stimulation, both biotic column systems were able to degrade a maximum of ~ 33 mg TCE/(L day) per column, only limited by the available oxygen. With additional electrochemical biostimulation, the degradation capability of the stimulated column was enhanced to ~ 50 mg TCE/(L day), which equals the supplied amount of TCE.

In summary, the study shows:

- Concentrations of up to 30 mg/L TCE can be treated in a flow-through bioreactor system with a retention time of 0.24 days.
- Low-strength DC can be applied to increase available oxygen in the reactor system and therefore increase the degradation performance in oxygen saturated as well as oxygen-deprived scenarios.
- The investigated DC strengths did not harm the established microbiota, resulting in a stable treatment process.

Only low current densities are required to stimulate the aerobic biodegradation of TCE. Adjusting the amount of produced oxygen can easily be done by altering the applied current strength. For a more sustainable remediation, renewable energy sources could be used to drive the process.

The study performed serves as a proof of concept for the electrochemical support of aerobic metabolic TCE degradation. Further laboratory studies to demonstrate the viability of the process under field relevant conditions with groundwater containing a mixture of contaminants need to be performed.

## Data Availability

Not applicable.

## References

[CR1] Arcangeli J-P, Arvin E (1995). A membrane de-oxygenator for the study of anoxic processes. Water Res.

[CR2] ATSDR (2019): Substance Priority List. ATSDR - Agency for Toxic Substances & Disease Registry. https://atsdr.cdc.gov/SPL/#2019spl, checked on 11/11/2022.

[CR3] Aulenta F, Verdini R, Zeppilli M, Zanaroli G, Fava F, Rossetti S, Majone M (2013). Electrochemical stimulation of microbial cis-dichloroethene (cis-DCE) oxidation by an ethene-assimilating culture. N Biotechnol.

[CR4] Bradley PM (2003). History and ecology of chloroethene biodegradation. A review. Bioremediat J.

[CR5] Cai Q, Shi C, Yuan S, Tong M (2022) Integrated anaerobic–aerobic biodegradation of mixed chlorinated solvents by electrolysis coupled with groundwater circulation in a simulated aquifer // Integrated anaerobic-aerobic biodegradation of mixed chlorinated solvents by electrolysis coupled with groundwater circulation in a simulated aquifer. Environ Sci Pollut Res Int. 10.1007/s11356-022-24377-8 10.1007/s11356-022-24377-8 36445524

[CR6] Conrad ME, Brodie EL, Radtke CW, Bill M, Delwiche ME, Lee MH (2010). Field evidence for co-metabolism of trichloroethene stimulated by addition of electron donor to groundwater. Environ Sci Technol.

[CR7] Dey K, Roy P (2009). Degradation of trichloroethylene by Bacillus sp. isolation strategy, strain characteristics, and cell immobilization. Curr Microbiol.

[CR8] Gaza S, Schmidt KR, Weigold P, Heidinger M, Tiehm A (2019) Aerobic metabolic trichloroethene biodegradation under field-relevant conditions. Water Res 343–348. 10.1016/j.watres.2018.12.022 10.1016/j.watres.2018.12.022 30616046

[CR9] Hazen TC, Chakraborty R, Fleming JM, Gregory IR, Bowman JP, Jimenez L (2009). Use of gene probes to assess the impact and effectiveness of aerobic in situ bioremediation of TCE. Arch Microbiol.

[CR10] Hyldegaard BH, Ottosen LM, Alshawabkeh AN (2020). Transformation of tetrachloroethylene in a flow-through electrochemical reactor. Sci Total Environ.

[CR11] IFA (2023): GESTIS-Substance Database. Trichloroethylene. Institut für Arbeitsschutz der Deutschen Gesetzlichen Unfallversicherung (IFA). https://gestis.dguv.de/data?name=010720&lang=en, checked on 8/2/2023.

[CR12] Illman WA, Alvarez PJ (2009). Performance assessment of bioremediation and natural attenuation. Crit Rev Environ Sci Technol.

[CR13] Lai A, Aulenta F, Mingazzini M, Palumbo MT, Papini MP, Verdini R, Majone M (2017) Bioelectrochemical approach for reductive and oxidative dechlorination of chlorinated aliphatic hydrocarbons (CAHs). Chemosphere 169: 351–360. 10.1016/j.chemosphere.2016.11.072 10.1016/j.chemosphere.2016.11.072 27886537

[CR14] Lohner ST, Becker D, Mangold K-M, Tiehm A (2011). Sequential reductive and oxidative biodegradation of chloroethenes stimulated in a coupled bioelectro-process. Environ Sci Technol.

[CR15] Lyon DY, Vogel TM, Stroo HF, Andrea Leeson C, Ward H (2013). Bioaugmentation for groundwater remediation. An overview. Bioaugmentation for Groundwater Remediation.

[CR16] Mattes TE, Alexander AK, Coleman NV (2010). Aerobic biodegradation of the chloroethenes. Pathways, enzymes, ecology, and evolution. FEMS Microbiol Rev.

[CR17] Rajic L, Fallahpour N, Alshawabkeh AN (2015). Impact of electrode sequence on electrochemical removal of trichloroethylene from aqueous solution. Appl Catal B, Environ.

[CR18] Rajic L, Nazari R, Fallahpour N, Alshawabkeh AN (2016). Electrochemical degradation of trichloroethylene in aqueous solution by bipolar graphite electrodes. J Environ Chem Eng.

[CR19] Ryoo D, Shim H, Canada K, Barbieri P, Wood TK (2000). Aerobic degradation of tetrachloroethylene by toluene-o-xylene monooxygenase of Pseudomonas stutzeri OX1. Nat Biotechnol.

[CR20] Sayavedra-Soto LA, Gvakharia B, Bottomley PJ, Arp DJ, Dolan ME (2010). Nitrification and degradation of halogenated hydrocarbons—a tenuous balance for ammonia-oxidizing bacteria. Appl Microbiol Biotechnol.

[CR21] Schmidt KR, Gaza S, Voropaev A, Ertl S, Tiehm A (2014). Aerobic biodegradation of trichloroethene without auxiliary substrates. Water Res.

[CR22] Shingleton JT, Applegate BA, Baker AJ, Sayler GS, Bienkowski PR (2001). Quantification of toluene dioxygenase induction and kinetic modeling of TCE cometabolism by Pseudomonas putida TVA8. Biotechnol Bioeng.

[CR23] Tiehm A, Schmidt KR (2011). Sequential anaerobic/aerobic biodegradation of chloroethenes—aspects of field application. Curr Opin Biotechnol.

[CR24] Tiehm A, Schmidt KR, Pfeifer B, Heidinger M, Ertl S (2008). Growth kinetics and stable carbon isotope fractionation during aerobic degradation of cis-1,2-dichloroethene and vinyl chloride. Water Res.

[CR25] Verdini R, Aulenta F, de Tora F, Lai A, Majone M (2015). Relative contribution of set cathode potential and external mass transport on TCE dechlorination in a continuous-flow bioelectrochemical reactor. Chemosphere.

[CR26] Willmann A, Trautmann A-L, Kushmaro A, Tiehm A (2023). Intrinsic and bioaugmented aerobic trichloroethene degradation at seven sites. Heliyon.

[CR27] Yuan S, Liu Y, Zhang P, Tong M, Liu H (2021). Electrolytic groundwater circulation well for trichloroethylene degradation in a simulated aquifer. Sci China Technol Sci.

[CR28] Zeppilli M, Dell’Armi E, Cristiani L, Petrangeli Papini M, Majone M (2019). Reductive/oxidative sequential bioelectrochemical process for perchloroethylene removal. Water.

[CR29] Zhao H-P, Schmidt KR, Tiehm A (2010). Inhibition of aerobic metabolic cis-1,2-di-chloroethene biodegradation by other chloroethenes. Water Research.

